# Evaluation of Homogentisic Acid, a Prospective Antibacterial Agent Highlighted by the Suitability of Nitisinone in Alkaptonuria 2 (SONIA 2) Clinical Trial

**DOI:** 10.3390/cells12131683

**Published:** 2023-06-21

**Authors:** Nicola Ooi, Ian R. Cooper, Brendan Norman, James A. Gallagher, Nick Sireau, George Bou-Gharios, Lakshminarayan R. Ranganath, Victoria J. Savage

**Affiliations:** 1 Infex Therapeutics, Alderley Park, Macclesfield SK10 4TG, UK; 2 Institute of Life Course and Medical Sciences, William Henry Duncan Building, University of Liverpool, Liverpool L7 8TX, UK; 3 AKU Society, Cambridge CB1 2BL, UK; 4 Departments of Clinical Biochemistry and Metabolic Medicine, Liverpool University Hospitals NHS Foundation Trusts, Liverpool L7 8XP, UK

**Keywords:** alkaptonuria, AKU, homogentisic acid, antimicrobial, bacteria, safety, mechanism of action

## Abstract

Despite urgent warnings about the spread of multidrug-resistant bacteria, the antibiotic development pipeline has remained sparsely populated. Naturally occurring antibacterial compounds may provide novel chemical starting points for antibiotic development programs and should be actively sought out. Evaluation of homogentisic acid (HGA), an intermediate in the tyrosine degradation pathway, showed that the compound had innate activity against Gram-positive and Gram-negative bacteria, which was lost following conversion into the degradation product benzoquinone acetic acid (BQA). Anti-staphylococcal activity of HGA can be attributed to effects on bacterial membranes. Despite an absence of haemolytic activity, the compound was cytotoxic to human HepG2 cells. We conclude that the antibacterial activity and in vitro safety profile of HGA render it more suitable for use as a topical agent or for inclusion in a small-molecule medicinal chemistry program.

## 1. Introduction

The true burden of antimicrobial-resistant (AMR) bacteria has long been speculated. Predictions that 10 million deaths could be associated with AMR bacteria by 2050 are likely to be a significant underestimation [1]. In 2019, approximately 5 million deaths were associated with antibiotic-resistant bacteria, of which 1.27 million deaths were directly attributable to infections caused by resistant organisms [2]. Therefore, it is imperative that new classes of antibiotics are developed to enable treatment of infections caused by multidrug-resistant bacteria. One of the greatest challenges to antibiotic development is ensuring the safety of compounds in humans. The cost to developers for progressing a small-molecule antibiotic from early-stage preclinical development to Phase I clinical trials is estimated to be up to EUR 12.5 million [3]; this is the first opportunity to fully assess safety and tolerability in humans. Development of chemical series derived from compounds that are already known to be tolerated in the body and have antibacterial activity may mitigate the risk of product failure due to drug-related adverse events during clinical trials. Here, we report the antibacterial activity, mode of action and in vitro toxicity of homogentisic acid (HGA), a naturally occurring molecule and intermediate in the tyrosine catabolic pathway [4].

The ultrarare human autosomal recessive disorder alkaptonuria (AKU) is characterised by the absence of homogentisate 1,2-dioxygenase enzyme activity, resulting in overproduction of HGA. Mean circulating concentrations of HGA reach 30.4 ± 0.8 µmol/L in AKU patients in comparison with 2.24 ± 0.25 µmol/L in non-AKU subjects [5]. Accumulation of HGA causes darkening of the urine, pigmentation and weakening of connective tissue and associated conditions, such as arthritis [6].

In 2020, following completion of the Suitability of Nitisinone in Alkaptonuria 2 (SONIA 2) clinical trial to assess the efficacy of nitisinone in the treatment of AKU, the European Medicines Agency approved nitisinone 10 mg once daily as the first disease-modifying, HGA-lowering therapy. The 4-year parallel-group study, described in detail by Ranganath et al. [7], demonstrated efficacy in AKU but also reported unexplained increases in infection rates in the nitisinone-treated group when compared to the placebo group (69 patients per group). There were 56 infection adverse events reported in 27 nitisinone-treated patients, while in the control group, there were 24 infection adverse events reported in 11 patients. Viral, bacterial and fungal infections were more frequently seen in the nitisinone group where urinary tract infections, pneumonia and bronchitis were more common than in the control group [7].

Nitisinone has been used to treat hereditary tyrosinaemia type 1 (HT-1) since 1991 [8]. No increased infection risk has been described either in PubMed or by the pharma company marketing nitisinone through its post-marketing surveillance; this argues against nitisinone itself causing increased infection rates, as the doses used in HT-1 are substantially higher than those used in SONIA 2 [9]. This suggests that HGA, only raised in AKU but not in HT-1, could be involved in reducing infections during SONIA 2. In support of this hypothesis, early studies identified antimicrobial properties of HGA at high concentrations [10], though antibacterial activity was not fully characterised, an omission that this study aims to address.

## 2. Materials and Methods

### 2.1. Bacterial Strains, Growth Media and Chemicals

*Staphylococcus aureus*, *Staphylococcus epidermidis* and *Haemophilus influenzae* strains were purchased from ATCC (via LGC Standards, Middlesex, UK). *Escherichia coli* N43 and the efflux defective derivative W4573 were obtained from the *E. coli* Genetic Stock Center (Yale University, New Haven, CT, USA). *Pseudomonas aeruginosa* PAO1 and the efflux defective derivative PAO750 were provided by Uppsala University (Uppsala, Sweden). Bacteria used in this study were propagated in cation-adjusted Mueller Hinton broth (CA-MHB) and on Mueller Hinton agar (MHA), or in Haemophilus testing medium broth (HTMB) and agar (HTMA) in the case of *H. influenzae*. Human HepG2 cells (ATCC^®^ HB-8065^TM^) were purchased via LGC Standards and were propagated in Minimum Essential Medium supplemented with 10% heat-inactivated fetal bovine serum and 1 mM sodium pyruvate.

Antibiotics and chemicals were from Sigma-Aldrich (Poole, UK) with the exception of cetyltrimethylammonium bromide, purchased from Cambridge Bioscience (Cambridge, UK), Opti-MEM™ and Triton X-100 from ThermoFisher Scientific (Waltham, MA, USA). BQA was isolated from HGA using the nitrous acid method [4]. Briefly, 0.1 mL of 1% NaNO_2_ was added to 0.1 mL of aqueous HGA (0.1 M). The solution was combined with 2 mL of phosphate-buffered saline (PBS; pH 6.8) and 0.5 mL of HCl (0.1 M), followed by incubation at room temperature for 5 min. A further 2.3 mL of PBS (pH 6.8) was added, and the solution was adjusted to pH 6.7. Conversion from HGA to BQA was confirmed spectrophotometrically (absorbance maxima 290 nm and 250 nm, respectively).

### 2.2. HGA Stability Studies

Solutions of homogentisic acid were prepared in PBS adjusted to pH 4, pH 5, pH 6, pH 7, pH 8 or pH 9. Solutions were incubated at 37 °C for 24 h and optical density measured at regular intervals using a FLUOstar Omega plate reader (BMG Labtech, Aylesbury, UK). The presence of HGA and BQA was detected at their absorbance maxima. Extinction coefficients were determined using known concentrations of HGA and BQA in PBS, and the Beer–Lambert law was used to calculate the concentration of compound present in solution over time [4].

### 2.3. Antibacterial Activity and Mechanism of Action

Minimum inhibitory concentrations (MICs) were determined via broth microdilution in 96-well plates according to CLSI guidelines [11]. In brief, a 10-point twofold dilution series of compound was prepared in CA-MHB for all organisms, with the exception of *H. influenzae*, for which HTMB was used. Bacterial colonies were suspended in broth and combined with compound or vehicle in wells at a final cell density of 5 × 10^5^ CFU/mL. Plates were incubated at 35 ± 2 °C for 20 h, following which MICs were determined by visual inspection. MICs were defined as the lowest concentration of compound that inhibits bacterial growth. Minimum bactericidal concentrations (MBCs) were determined following challenge with concentrations of compounds above the MIC. Bacteria were serially diluted, spread on appropriate agar and colonies were counted after incubation at 37 °C for 18–24 h. The MBC was defined as the minimum concentration of antibacterial agent that caused a 99.9% kill [12].

The effects of compounds on bacterial membrane integrity were assessed using the Live/Dead BacLight^TM^ kit (ThermoFisher Scientific) following exposure of *S. aureus* ATCC 29213 to compounds at multiples of the MIC for 10 min [13]. Exponential phase cultures of bacteria (OD_600_ 0.5–0.6) were washed in sterile deionised water and resuspended to twice the volume. Cells were incubated with 5% SDS or test compounds at multiples of the MIC for 10 min at room temperature with aeration, then washed and resuspended in water. Equal volumes of BacLight^TM^ component A and component B were mixed and diluted 1:300 in water, and 150 µL was combined with 50 µL of cells in a black 96-well plate. Following incubation in the dark for 15 min at room temperature, fluorescence was measured at an excitation of 485 nm and emission of 520 nm or 620 nm using a FLUOstar Omega plate reader. The ratio of green/red fluorescence was determined, and percentage loss of membrane integrity was calculated with respect to the SDS control, with >30% loss considered a significant effect. The effects of compounds on mammalian membranes were examined by measuring lysis of human erythrocytes. Lithium heparin-treated whole blood (Cambridge Bioscience) was centrifuged at 3000× *g* for 5 min at 4 °C and the supernatant and buffy coat discarded. The erythrocyte pellet was washed twice and resuspended in PBS (pH 7.4) at 4 °C. Erythrocytes were diluted in PBS (pH 7.4) prewarmed to 37 °C (1 × 10^8^ cells/mL) and exposed to compounds at 128 or 512 µg/mL for 1 h, with mixing by inversion every 20 min. Cell suspensions were centrifuged at 3000× *g* for 5 min, and the extent of haemoglobin leakage was measured in the supernatant at OD_540_ [14]. Percentage haemolysis was calculated relative to 0.1% Triton X-100.

### 2.4. In Vitro Toxicity

Cytotoxicity of compounds was evaluated in human HepG2 cells (ATCC^®^ HB-8065^TM^) seeded at a density of 2 × 10^4^ cells/well in a white 96-well plate and incubated for 24 h at 37 °C and 5% CO_2_. Cells were washed in Dulbecco’s PBS then exposed to compounds at 512 µg/mL in Opti-MEM^TM^ for 24 h before determination of viability using the CellTiter-Glo^®^ kit (Promega, WI, USA) according to the manufacturer’s instructions [15]. As such, treated cells were washed in Dulbecco’s PBS then incubated at room temperature for 10 min with CellTiter-Glo^®^ reagent (diluted 1:5 in Dulbecco’s PBS). Luminescence was detected using a FLUOstar Omega plate reader, and percentage loss of viability was calculated relative to the vehicle control. A dilution series of chlorpromazine was used as an internal positive control.

## 3. Results

When exploring the biological activity of metabolites, such as HGA, it is essential that their stability is assessed, since they undergo conversion to other chemical species, which may have undetermined properties. In solution, HGA degrades, forming a dark-coloured product; previous research indicated that progression of pigment formation is dependent upon pH of the solution, and formation of the primary degradation product, BQA, may be prevented at low pH [16]. Therefore, the stability of HGA was studied spectrophotometrically at discrete pH values ranging from 4 to 9. An HGA concentration of 512 µg/mL allowed for maximum sensitivity of detection; thus, stability studies were carried out over 24 h at 512 µg/mL in PBS. No significant degradation of HGA and concomitant formation of BQA were identified in PBS adjusted to pH 4, pH 5 or pH 6. Limited formation of BQA was identified by 3 h at pH 7 and significant formation by 1 h at pH 8 and pH 9 (Figure S1). This confirms that HGA degradation occurs more rapidly, and extensively, at and above neutral pH, as has been suggested previously [4,16], indicating that adjustment of culture media pH may be used to evaluate antibacterial activity at various states of HGA degradation.

*S. aureus* is responsible for one in seven deaths attributed to AMR bacteria [2]. The highly cited 2017 WHO priority pathogen list classified methicillin- and vancomycin-resistant *S. aureus* as a high priority for future antibiotic discovery programs [17]. Consequently, initial studies interrogated the relationship between the stability of HGA and antibacterial activity using *S. aureus* ATCC 29213. MICs were performed in CA-MHB adjusted to pH 5–pH 9, the range at which *S. aureus* remained viable. In culture media, no pigment was formed at pH 6 or below following 20 h incubation at 37 °C, whilst increasing pigment formation was identified as pH increased, a predictable observation given the spectral studies performed in PBS. MIC determinations revealed an inverse relationship between pigment formation and antibacterial activity (Table 1). At pH 5, HGA had mild antibacterial activity against *S. aureus* ATCC 29213 (MIC 2048 µg/mL), which was lost at higher pH, suggesting that HGA degradation and concurrent appearance of BQA (or subsequent degradants) were associated with a loss of antibacterial activity. In the absence of commercially available BQA, material was isolated from HGA. BQA displayed no antibacterial activity at the maximum testable concentration (MIC > 32 µg/mL). Collectively, degradation studies and *S. aureus* susceptibility testing indicate that HGA has innate antibacterial activity, while degradation products do not. As such, further characterisation was performed with HGA.

Antibiotic-resistant Gram-negative pathogens are associated with increased rates of empirical antibiotic therapy failure and worse patient outcomes [18]. Indeed, *E. coli* is the leading cause of deaths attributable to antibiotic-resistant bacterial infections [2]. Therefore, standard susceptibility testing was performed using an extended panel of clinically relevant Gram-negative and Gram-positive pathogens, including *E. coli* (Table 1). HGA had modest antibacterial activity against all organisms with the exception of *P. aeruginosa* PAO1, for which there was no measurable MIC. The efflux defective derivative, *P. aeruginosa* PAO750 (Δ*mexAB-oprM*, Δ*mexCD-oprJ*, Δ*mexEF-oprN*, Δ*mexJK*, Δ*mexXY*, Δ*opmH*, Δ*pscC*), was inhibited by HGA (MIC 4096 µg/mL), suggesting that efflux of the compound contributes to the lack of activity against *P. aeruginosa* PAO1. However, extrusion of HGA is not responsible for reduced activity in all species, as efflux defective *E. coli* N43 (Δ*acrA*) had equivalent susceptibility to the efflux proficient parent *E. coli* W4573. Although HGA had measurable MICs in a range of species, bactericidal activity was not identified in this strain panel during MBC determinations (MBC > 4096 µg/mL).

To investigate the mechanism by which HGA exerts antimicrobial activity, the effect of the compound on bacterial membranes was studied. *S. aureus* ATCC 29213 membrane integrity was assessed following exposure to control compounds and HGA for 10 min. The negative controls tetracycline and ciprofloxacin (protein synthesis inhibitor and DNA synthesis inhibitor, respectively) caused no significant disruption of the staphylococcal membrane at 4 × MIC (Table 2). Exposure to HGA at the MIC or sub-MIC concentrations resulted in loss of membrane integrity, the extent of which was comparable with that of the positive control, CTAB. Therefore, data from this assay indicate that HGA is a rapidly acting membrane-damaging agent that is capable of killing bacteria within 10 min. This mechanism of action is consistent with clinical trial findings, in which an excess of HGA appeared to reduce the frequency of bacterial, viral and fungal infections [7]. To explore the specificity of membrane interactions, the effects on mammalian membranes were evaluated by monitoring haemolysis of human erythrocytes. Although sub-inhibitory concentrations of HGA caused an 80% reduction in staphylococcal membrane integrity, haemolysis was not induced at this concentration (Table 2). This suggested that HGA may display some selectivity for bacterial membranes, as is the case for outer-membrane-disrupting antibiotics such as the control compound colistin.

Despite significant accumulation of HGA (4.86 ± 0.13 g/day) in AKU patients involved in the SONIA 2 clinical trial, circulating concentrations of only 30 µmol/L were reached due to extremely efficient renal clearance. The daily amount of pure HGA eliminated from the body in the urine of AKU patients was 30,376 ± 837 µmol/L, equating to 5108 µg/mL [5]. Therefore, human tissues are exposed to and appear to tolerate a concentration of HGA above that required to inhibit organisms, such as *E. coli* and staphylococci (MIC 4096 µg/mL). To test the safety assumptions drawn from this clinical observation, the effect of HGA on cultured human cells was determined. The bacterial outer membrane permeabiliser colistin was non-cytotoxic at 128 µg/mL (512 × *E. coli* MIC). However, HGA was cytotoxic at 512 µg/mL, causing total loss of human cell viability at a concentration below the bacterial MIC (Table 2). This level of cytotoxicity at a concentration that was not haemolytic likely reflects the more robust nature of erythrocytes and duration of HepG2 exposure to HGA (1 h and 24 h for haemolysis and cytotoxicity assays, respectively).

## 4. Discussion

Although the SONIA 2 clinical trial indicated that HGA should be evaluated as part of an antibiotic discovery program, early promise was not borne out during in vitro microbiological and safety evaluations. This serves as a reminder that it is necessary to carry out early in vitro feasibility studies, even when the compound under investigation is naturally occurring in humans. The presence of modest antibacterial activity at HGA concentrations that induce cytotoxic effects in cultured human cells indicates that the compound does not display specific inhibition of bacteria. Furthermore, as HGA is converted to BQA more quickly under alkaline conditions, such as those found in the small intestine, orally dosed HGA would likely not be absorbed sufficiently to reach the systemic circulation at concentrations that exert antibacterial effects. Modifying the HGA molecule to increase its potency and bioavailability in order to attain safe circulating concentrations has not yet been explored. In an alternative approach, HGA could be utilised via innovative gene silencing techniques, employing a transient knock down of the homogentisate 1,2-dioxygenase gene in vivo using siRNA, temporarily increasing HGA systemically during an acute infection. Proof of concept for the use of antisense oligonucleotides as antimicrobial therapies was first achieved for the treatment of cytomegalovirus retinitis [19]. Therefore, research to assess the feasibility of this novel approach could complement a traditional drug development program.

In view of the data presented here, HGA is not a viable candidate for development as a systemic antibiotic, its properties rendering the compound more appropriate for use as a topical antimicrobial agent if used in its current form. There is a precedent for the use of membrane-damaging antimicrobials in healthcare products, such as 1% silver sulfadiazine cream, for the treatment of burn wounds. When applied at high concentrations at the site of infection, compounds that disrupt bacterial membranes are less likely to select for highly drug-resistant organisms and circumvent any potential systemic tolerability liabilities, as is the case for silver sulfadiazine [20].

## 5. Conclusions

In this study, HGA was proven to have innate antimicrobial activity, resulting from interaction with the bacterial cell membrane. Due to the potency and safety profile of HGA, it may be most suited to development as a topical antimicrobial agent or serve as a starting point for a medicinal chemistry program to develop new antimicrobials from this naturally occurring metabolite.

## Figures and Tables

**Table 1:** Minimum inhibitory concentrations of HGA and a comparator antibiotic against Gram-positive and Gram-negative bacteria.

Organism [pH]	MIC (µg/mL)	
	Homogentisic Acid	Ciprofloxacin
*Staphylococcus aureus* ATCC 29213 [pH 5]	2048	nd
*Staphylococcus aureus* ATCC 29213 [pH 6]	4096	nd
*Staphylococcus aureus* ATCC 29213 [pH 7]	4096	nd
*Staphylococcus aureus* ATCC 29213 [pH 8]	4096	nd
*Staphylococcus aureus* ATCC 29213 [pH 9]	>4096	nd
*Staphylococcus aureus* ATCC 29213	4096	0.25
*Escherichia coli* W4573	4096	0.03
*Escherichia coli* N43 *	4096	0.004
*Staphylococcus epidermidis* ATCC 12228	4096	0.25
*Pseudomonas aeruginosa* PAO1	>4096	0.5
*Pseudomonas aeruginosa* PAO750 *	4096	0.03
*Haemophilus influenzae* ATCC 49247	2048	0.008

* *E. coli* and *P. aeruginosa* strains are isogenic pairs: N43 is an efflux defective derivative of W4573; PAO750 is an efflux defective derivative of PAO1. Where pH is not specified, MICs were performed in commercial CA-MHB (pH 7.4). nd, no data. Values were determined from at least two independent replicates.

**Table 2:** *S. aureus* and human cell integrity following exposure to HGA and control antibiotics at multiples of the MIC.

Compound	Concentration (µg/mL) [×MIC]	% Loss *S. aureus* * Membrane Integrity (±SD)	% Haemolysis (±SD)	% Loss HepG2 Viability (±SD)
Tetracycline	8 [4 × MIC *]	11.0 ± 9.2	nd	nd
Ciprofloxacin	1 [4 × MIC *]	21.6 ± 7.2	nd	nd
CTAB	4 [4 × MIC *]	81.0 ± 4.2	nd	nd
Homogentisic acid	4096 [1 × MIC *]	81.2 ± 4.4	nd	nd
Homogentisic acid	512 [0.125 × MIC *^#^]	79.9 ± 3.7	−0.7 ± 0.6	99.6 ± 0.1
Colistin	128 [512 × MIC ^#^]	nd	16.1 ± 4.0	−13.9 ± 15.9

* *S. aureus* ATCC 29213; ^#^ *E. coli* W4573; CTAB, cetyltrimethyl ammonium bromide; nd, no data; SD, standard deviation. Values were determined from at least four replicates.

## Data Availability

All data analysis is presented in this published article. The datasets generated during the current study are available from the corresponding author upon request.

## Supplementary Materials

The following supporting information can be downloaded at: https://www.mdpi.com/article/10.3390/cells12131683/s1, Figure S1: Formation of BQA during incubation of HGA in PBS at pH 4–pH 9.
