# Supplementary material for: Evaluation of Homogentisic Acid, a Prospective Antibacterial Agent Highlighted by the Suitability of Nitisinone in Alkaptonuria 2 (SONIA 2) Clinical Trial
Source: Cells. 2023 Jun 21;12(13):1683. doi: 10.3390/cells12131683 (PMC10341174; doi:10.3390/cells12131683)
Supplement: Supplementary file 1 [file cells-12-01683-s001.zip › cells-2404850-supplementary.pdf]

Evaluation of homogentisic acid, a prospective antibacterial agent highlighted by the Suitability of Nitisinone in Alkaptonuria 2 (SONIA 2) clinical trial

Supplementary Materials

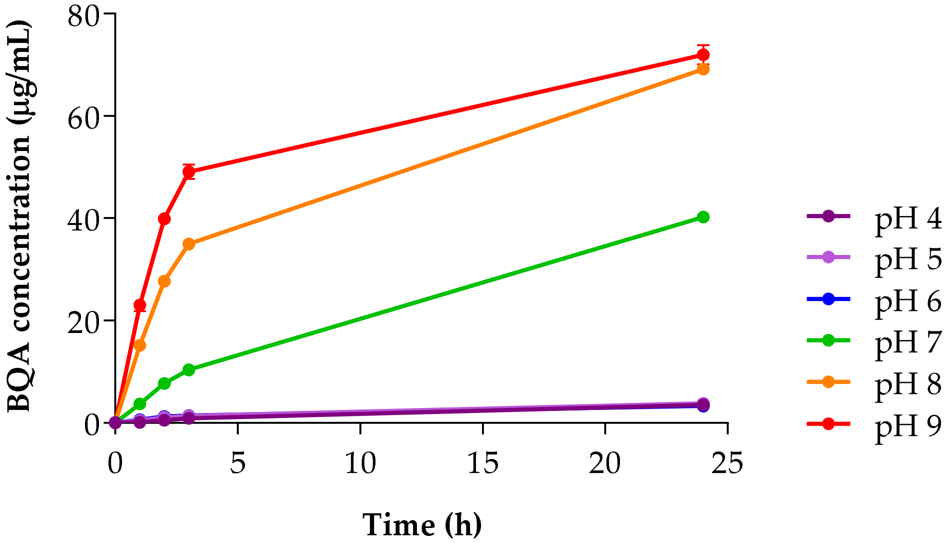

Figure S1. Formation of BQA during incubation of HGA in PBS at pH 4 – pH 9. Values were determined from at least two independent replicates; error bars indicate maximum and minimum values.
